# Chromosome X-wide Analysis of Positive Selection in Human Populations: Common and Private Signals of Selection and its Impact on Inactivated Genes and Enhancers

**DOI:** 10.3389/fgene.2021.714491

**Published:** 2021-09-27

**Authors:** Pablo Villegas-Mirón, Sandra Acosta, Jessica Nye, Jaume Bertranpetit, Hafid Laayouni

**Affiliations:** ^1^ Institut de Biologia Evolutiva (UPF-CSIC), Universitat Pompeu Fabra, Barcelona, Spain; ^2^ Department Pathology and Experimental Therapeutics, Medical School, University of Barcelona, Barcelona, Spain; ^3^ Bioinformatics Studies, ESCI-UPF, Barcelona, Spain

**Keywords:** X-chromosome, positive selection, human populations, hard and soft sweeps, enhancers

## Abstract

The ability of detecting adaptive (positive) selection in the genome has opened the possibility of understanding the genetic basis of population-specific adaptations genome-wide. Here, we present the analysis of recent selective sweeps, specifically in the X chromosome, in human populations from the third phase of the 1,000 Genomes Project using three different haplotype-based statistics. We describe instances of recent positive selection that fit the criteria of hard or soft sweeps, and detect a higher number of events among sub-Saharan Africans than non-Africans (Europe and East Asia). A global enrichment of neural-related processes is observed and numerous genes related to fertility appear among the top candidates, reflecting the importance of reproduction in human evolution. Commonalities with previously reported genes under positive selection are found, while particularly strong new signals are reported in specific populations or shared across different continental groups. We report an enrichment of signals in genes that escape X chromosome inactivation, which may contribute to the differentiation between sexes. We also provide evidence of a widespread presence of soft-sweep-like signatures across the chromosome and a global enrichment of highly scoring regions that overlap potential regulatory elements. Among these, enhancers-like signatures seem to present putative signals of positive selection which might be in concordance with selection in their target genes. Also, particularly strong signals appear in regulatory regions that show differential activities, which might point to population-specific regulatory adaptations.

## Introduction

The evolution of *Homo sapiens* has been strongly shaped by positive selection in the last 100,000 years, by adapting to specific environments, diets, and cognitive challenges as populations expanded across the globe. Surviving such challenges has left remarkable footprints of selection in the human genome, like in the lactase (*LCT*) locus in European populations (Bersaglieri et al., 2004; Wang et al., 2020), genes involved in skin pigmentation like *MC1R* (John et al., 2003) or genes implicated in resistance to severe malaria infection like *CD40L* and *G6PD* (Sabeti et al., 2002). Studying the evolutionary processes that resulted from these adaptations can uncover which path our ancestors travelled along to give rise to extant adaptations of present human populations.

The development of new methods to study recent selection in natural populations (Fan et al., 2016; Field et al., 2016; Pavlidis and Alachiotis, 2017) has allowed for these adaptations to be assessed by genomic selection scans (Mathieson et al., 2015; Casillas et al., 2018; Lopez et al., 2019; Walsh et al., 2020). However, most of these scans have focused on coding regions as the main target of selection and focused on processes involving *de novo* mutations which leave strong and more evident selection signatures (classical hard sweeps). Although gene regulation is considered to be the primary driver of phenotypic changes in the evolution of *Homo sapiens* (King and Wilson, 1975), selection on standing variation in regulatory regions may have been overlooked. Selection on standing variation seems to be the more likely target of rapid adaptation and is marked by more subtle signatures, like soft sweeps (Fu and Akey, 2013; Messer and Petrov, 2013; Scheinfeldt and Tishkoff, 2013).

This mode of selection is likely to be common among humans, leaving widespread signatures in human genomes (Hernandez et al., 2011; Schrider and Kern, 2017). The soft sweep signature may be caused by selection on standing variation, the *de novo* mutation on multiple haplotypes, and recurrent origination of adaptive alleles (Schrider et al., 2015; Hermisson and Pennings, 2017). These signatures exhibit different degrees of “softness” and, together with confounding factors like demography or recombination, display sweep-like signatures. In these cases, it is not clear enough to define a region as the target of a specific selection mode (Messer and Petrov, 2013). In addition, linked regions under selection often present properties of both types of signals (Schrider et al., 2015). Therefore, it is difficult to differentiate between hard and soft sweep signatures.

The X chromosome has been studied for evidence of recent positive selection (Casto et al., 2010; Veeramah et al., 2014; Johnson and Voight, 2018). However, selection on regulatory regions and standing variation have not been sufficiently assessed. The X and Y chromosomes have different inheritance models and effective population sizes than the autosomes, making the response to selection pressures differ from the rest of the genome. In order to study the X chromosome, these unique properties must be accounted for with chromosome-specific demographic models and region-specific recombination maps.

The particular properties of the X chromosome have been extensively studied (Vicoso and Charlesworth, 2006; Mank et al., 2010; Meisel and Connallon, 2013). Dosage compensation is the process which allows XY males and XX females to cope with differing gene copy numbers, and might lead to sex-specific patterns of selection. This process involves the random transcriptional silencing of one of the X chromosomes in females. However, the inactivation process is not complete for all the genes. Evidence suggests that around 23% of the X-linked genes “escape” inactivation and both chromosomal copies are expressed (Balaton et al., 2015; Tukiainen et al., 2017), leading to a sex-biased expression. Dimorphic traits and observed phenotypic diversity may be in part caused by sex-specific differential expression. Despite the paucity of data about selection on these genes, some evidence indicates these regions have been under stronger purifying selection than genes which do not escape X inactivation (Park et al., 2010). Thus, it is of interest to assess the relative importance of X inactivation in the process of natural selection.

The faster-X hypothesis (Meisel and Connallon, 2013) postulates that selection occurs more rapidly on the X than the autosomes due to the hemizygosity of males. This hypothesis has been supported by evidence of increased levels of selection on the X (Veeramah et al., 2014), by differing effects of mutations between genders (Vicoso and Charlesworth, 2006), and the difference in the sex-based germ line replication rate. The higher probability of suffering consequences due to deleterious and adaptive mutations most likely has led to a unique process of selection. Altogether, these factors may have led to patterns which reflect sex-biased evolution in humans.

In this study, we conduct a selection scan of the X chromosome among 15 populations from three continental groups (Sub-Saharan Africa, Europe and Asia). We sought to identify signatures of recent positive selection by searching for patterns of hard and soft sweeps in both coding and non-coding regions with the aim of disentangling how positive selection has shaped diversity across the globe.

## Materials and Methods

### Genetic Data

Phased VCF files from the third phase of the 1,000 Genomes Project were downloaded from the project database (Auton et al., 2015). These data are whole-genome (mean depth of 7.4X) and targeted exome sequences (mean depth of 65.7X) with a total of 2,504 individuals across 26 different populations. Due to methodological complexity, only the non-admixed populations of each geographical group were analyzed. We included the populations from Africa: Esan (Nigeria, ESN), Gambian (Wester Divisions in the Gambia, GWD), Luhya (Webuye, Kenya, LWK), Mende (Sierra Leone, MSL), Yoruba (Ibadan, Nigeria, YRI); Europe: Utah residents with northern and western European ancestry (CEU), Finnish (Finland, FIN), British (England and Scotland, GBR), Iberians (Spain, IBS), Toscani (Italy, TSI); and Asia: Chinese Dai, (Xishuangbanna, China, CDX), Han Chinese (Beijing, China, CHB), Southern Han Chinese (China, CHS), Japanese (Tokyo, Japan, JPT), Kinh (Ho Chi Minh City, Vietnam, KHV). We applied filters to remove duplicated variants reported to the 1,000 Genomes Project (www.1000genomes.org).

The X chromosome consists of both pseudoautosomal regions (PAR) and non-pseudoautosomal regions (nPAR). Since the PAR behaves differently and does not follow the same inheritance rules, we removed these regions keeping only bi-allelic variants of the nPAR region (∼2.7–155.0 Mb) (Flaquer et al., 2008).

We reformatted the VCF file so that the ancestral allele was the reference and the derived allele was the alternative. The human ancestral alleles determined by their state in chimpanzee were downloaded from the 1,000 Genomes Project mapped to human reference GRCh37. We removed any single nucleotide polymorphism (SNP) whose ancestral status was unknown, resulting in a total of 2,852,479 SNPs from 1,511 individuals (504 Africans, 503 Europeans, and 504 Asians).

We downloaded a population-combined genetic map of the nPAR region (http://mathgen.stats.ox.ac.uk). This map was based on the first phase of The 1,000 Genomes Project (GRCh37). In order to use the map for phase three data, we performed a linear interpolation of the missing values using the command *approx* from the statistical programming language R.^1^

### Neutral Simulations

We used the *msms* software (Ewing and Hermisson, 2010) to simulate neutral scenarios. For the X chromosome, we implemented a three-population demographic neutral model adapted from Henn et al. (2015) for the continental populations Africa (AFR), Europe (EUR), and Asia (ASI) with a mutation rate of 1.25x10⁻⁸ mutations per base per generation (Henn et al., 2015), a generation time of 30 years, a recombination rate of 1.3x10^−8^ per nucleotide, and a theta (*θ* = 4Neμ) of 328.79. We chose a three-population model due to the high similarity within continents, with a sample size obtained as the arithmetic mean from the five population sizes (number of chromosomes) in each continental group: Africa (ESN (145), GWD (171), LWK (154), MSL (128), YRI (164)), Europe (CEU (149), FIN (160), GBR (136), IBS (160), TSI (161)), and Asia (CDX (142), CHB (160), CHS (158), JPT (152), KHV (152)). This resulted in the following final sizes: AFR (152), EUR (153), and ASI (149). Since the effective population size of the X is ¾ the size of the autosomes, we accounted for this by modifying the population sizes, resulting in N_e_ for AFR: 23,220, EUR: 2,479, and ASI: 907. We simulated multiple regions of 600 kb in order to reproduce the total length of the X chromosome, by using the following parameters:

msms-N 10538.25-ms 454 254-t 316.1475-r 328.7934 600000-I 3 152 153 149 0-n 1 2.204-n 2 3.2542-n 3 7.4055-g 2 56.61-g 3 96-ma x 0.3542 0.1462 0.3542 x 1.3562 0.1462 1.3562 x-ej 0.0464 3 2-en 0.0464 2 0.2939-em 0.0464 1 2 4.9314-em 0.0464 2 1 4.9314-ej 0.14022 2 1-en 0.364 1 1-oTPi 30000 25000-tt-oAFS.

In order to contrast the results obtained for the X chromosome, we analyzed the complete set of autosomes in the human genome. The same procedure to detect positive selection as for the X was followed. To do so, we performed the appropriate autosomal neutral simulations and used the 99th percentile as extreme distribution cut-off to compare the regions under positive selection. Also, the Refseq gene annotation from the UCSC database table browser (Karolchik et al., 2004) (downloaded June 2020) was considered.

### Scan for Signals of Selection

Advances in statistics used to detect selective sweeps allow for the detection of linkage disequilibrium (LD) decay (Vallender, 2004; Biswas and Akey, 2006; Sabeti et al., 2006; Garud et al., 2015; Pybus et al., 2015). These methods detect decreased variation surrounded by a region with high LD, which increases as the surrounding variation decreases as the selected allele rises in frequency among the population. Once the selected allele is fixed, variation in the region is recovered through new mutations and recombination. The extended haplotype homozygosity (EHH) computes the probability that, at a given distance from a core region, two randomly chosen chromosomes carry homozygous SNPs for the entire interval. In this analysis, we use three different haplotype-based statistics which rely on the EHH at a tested SNP, taking into account the ancestral and derived allele state.

The integrated haplotype score (iHS) is the integral (Voight et al., 2006) of EHH and is designed to detect incomplete hard sweeps. These are signatures of recent, ongoing selection that are generated by the rise in frequency of the selected allele in a population, purging linked variation in the immediate region. This process generates long blocks of unbroken haplotypes at high frequency with a higher haplotype homozygosity in comparison with neutral regions. We have used two methods to detect signatures that resemble soft sweeps. The integrated haplotype homozygosity pooled (iHH12) (Torres et al., 2018) is an adaptation of the H12 statistic by Garud et al. (2015) and detects signatures of both hard and soft sweeps. The number of segregating sites by length (nSL) (Ferrer-Admetlla et al., 2014) is a modification of iHS which is more robust to recombination rate variation and has increased power to detect soft sweeps. These are the footprints left by selection processes which target intermediate frequency variants. Contrasted with hard sweeps which fixes a single *de novo* mutation, soft sweeps often target an allele neutrally drifting in the population (Orr and Betancourt, 2001; Hermisson and Pennings, 2005; Pennings and Hermisson, 2006). Also, these footprints of selection may occur when different alleles are selected simultaneously at the same locus. Therefore, the evidence of this kind of selection is more difficult to detect than hard sweeps because the genetic diversity is less impacted. These tests for recent positive selection are standardized (mean 0, variance 1) by the distribution of observed scores over a range of SNPs with similar derived allele frequencies. We use the three tests, iHS, nSL, and iHH12, to detect selective sweeps in the X chromosome.

**FIGURE 1:**
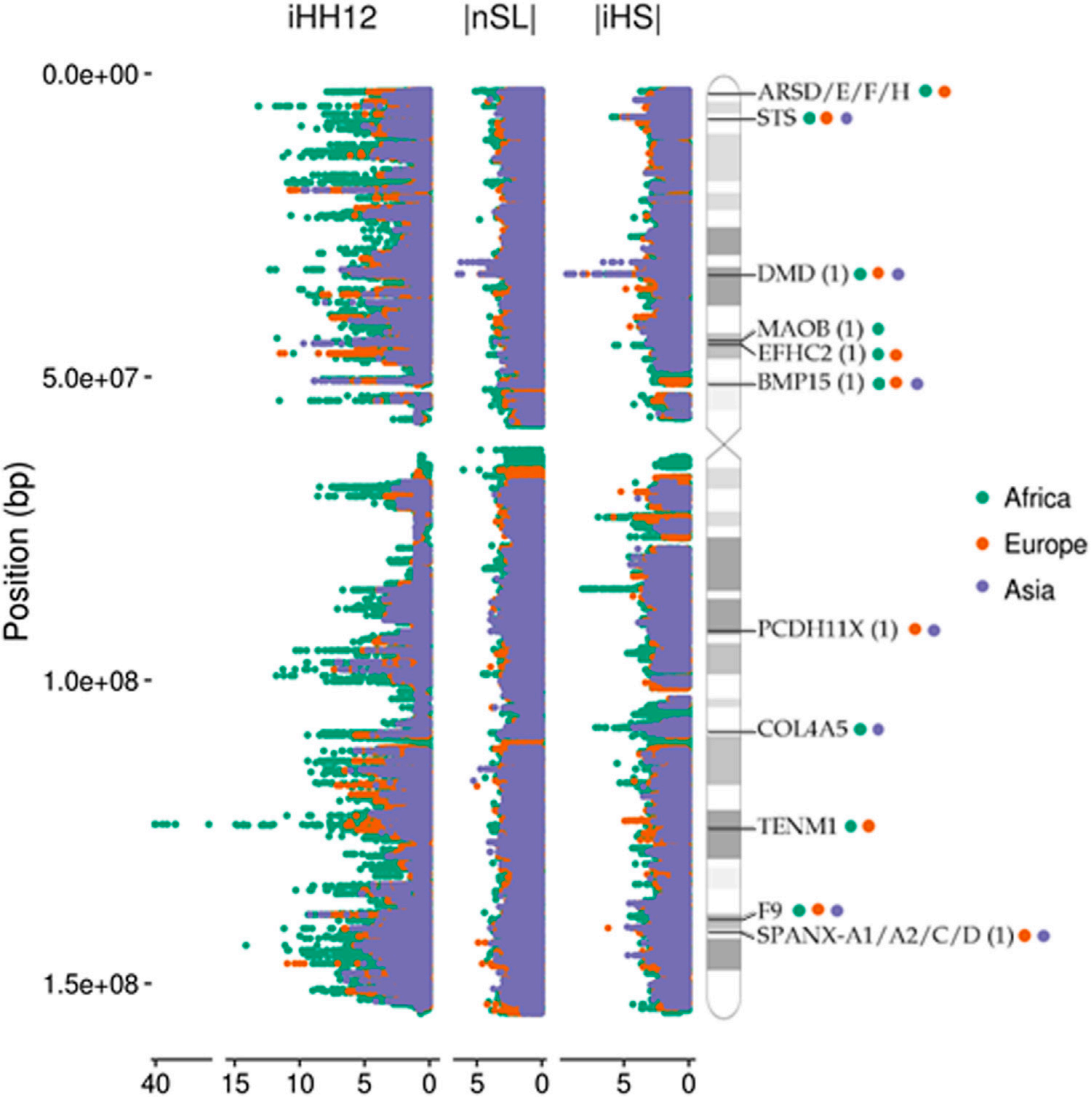
Manhattan plots of the X chromosome showing the distributions of the three selection tests used in the analysis. Some examples of genes found under selection in continental groups (99th percentile; coloured circles) are shown in the ideogram. Candidates found in previous studies are indicated with (1).

iHS, nSL, and iHH12 were computed with the software *selscan* (Szpiech and Hernandez, 2014). We allowed for a maximum gap of 20 kb and kept only SNPs with a minor allele frequency (MAF) > 5%. These parameters reduced the number of false positives due to gaps in the data. The same procedure was applied to the simulated data in order to compare the empirical distributions with a neutral scenario. Standardization was performed by the *norm* function within the *selscan* package for each population and statistic separately. Selection scores are represented in Figure 1 as Manhattan plots along the chromosome X ideogram obtained from the PhenoGram online tool (Wolfe et al., 2013).

The per-SNP scores were summarized using a position-based sliding window approach of size 20 kb with a 20% overlap (4 kb). Windows with 20 SNPs or fewer were removed. The mean scores were calculated in each test in order to interpret the presence or absence of a selective sweep. To search for candidate windows under positive selection, we compared the distributions of values to the simulations and considered 99th and 99.9th percentiles as critical values for evidence against neutrality. No *p*-values were associated with the significance of these windows.

Calculations of Tajima’s D were performed by using the software package *VCFtools* (0.1.14) with a non-overlapping 10 kb sized window-based approach (Danecek et al., 2011).

The haplotype structure in regions under putative positive selection was determined with the program *fastPHASE* (Scheet and Stephens, 2006)which applies a Hidden Markov Model (HMM) on haplotype data to obtain the frequencies of a certain SNP in a haplotype cluster, such that the region is divided into a mosaic of clusters per population and reflects the patterns of haplotypic variation.

In order to assess commonalities and differences across populations, we identified regions under selection in the extreme tail of more than one population. Since a region under positive selection can be captured by more than one test depending on the variable degree of “softness” in its locus, the shared regions were constructed by using the candidate windows reported in the extreme 99th percentile across the three selection tests. Sweeping regions that overlapped between multiple populations of the same continental group were considered shared in that group.

### Gene Ontology

We downloaded Refseq gene annotations from the UCSC database table browser (Karolchik et al., 2004) in June 2020. This annotation describes all the transcripts including 5′ and 3′ untranslated regions (UTR), coding, and non-coding genes. We merged these annotations with our empirical data using *Bedtools intersect* (Quinlan and Hall, 2010). We intersected our candidate windows under selection with the annotated genomic regions to obtain a list of genes under putative positive selection. Finally, an Overrepresentation Enrichment Analysis (OEA) was performed on the top 100 genes for each population with the online tool *WebGestalt GSAT* (Gene Set Analysis Toolkit). For each population and selection test, the top 100 genes were compared to a background constructed by the genic windows considered in the selection analysis. Multiple testing was adjusted using the Benjamini-Hochberg correction, accepting ontology terms with a global false discovery rate (FDR) ≤ 0.05 as significant. The same procedure was conducted on the pooled autosomal regions under positive selection when comparing with the sexual chromosomes.

In order to focus on putative regions with the highest selection scores, we selected the top windows that fall into the 99.9th percentile. The SNPs contained in these windows were annotated using the *ANNOVAR* program (Wang et al., 2010), which aggregates the UCSC annotations: GWAS Catalog, CADD scores, GERP++ scores, Conserved transcription factor binding sites (TFBS) in the human/mouse/rat alignment, segmental duplications, and clusters of TFBS based on ChIp-seq data. In order to identify the most interesting SNPs inside each region, we considered SNPs with an individual selection value within the 1% extreme tail of the distribution (|iHS| and |nSL| ∼ 2.5 in all populations, and iHH12 ∼ (Africa: 4.1, Europe: 3.8, and Asia: 3.6)) and a PHRED-scaled CADD score ≥10, representing the 1% most deleterious SNPs genome-wide (Kircher et al., 2014). To prioritize SNPs located in regulatory regions, we explored the potential effects of SNPs from both 99th and 99.9th top windows within functional regions by using RegulomeDB (Boyle et al., 2012). This database uses ENCODE data sets to annotate variants that are likely to belong to a functional region by scoring variants according to the amount of support to be functional elements. Category 1 corresponds with variants which are an expression quantitative trait loci (eQTL) and category 6 is a hit in a single motif.

### “Escape” Genes Selection Analysis

The X chromosome inactivation (XCI) status was determined using the catalog by Tukiainen et al. (2017), which includes a consensus of XCI statuses from previous studies (Carrel and Willard, 2005; Cotton et al., 2013) with additional data from the Genotype-Tissue Expression (GTEx) project (v6p release). The integrated statuses of these studies fall into three categories: *escape* (if “escape” and “variable”), *variable* (if “escape” and “inactive”), and *inactive* (if “variable” and “inactive”). Contingency tables were constructed based on selection (Selected/Not selected) and XCI (Escape/Inactive) statuses and assessed by a Fisher’s exact test.

### Regulatory Regions Under Positive Selection

The Human Active Enhancer to interpret Regulatory variants (HACER) database (Wang et al., 2019) was used to annotate intergenic windows in order to study potential signals of positive selection in enhancer-like regions. HACER annotates a total of 1,676,284 active enhancers (whole genome) detected by different methods (GRO-seq, PRO-seq and CAGE) in numerous cell lines and supported by different databases (VISTA, ENCODE Enhancer-like Regions, The Ensembl Regulatory Build and chromatin state segmentation by ChromHMM). In order to reduce the noise and provide a higher confidence to our intergenic signals, we used the 23,790 enhancers that are supported by database(s). In HACER, a given region can be annotated as an active enhancer in different cell lines, targeting the closest gene but presenting different coordinates. In order to deal with the different cell-type-specific annotations we created a “consensus” dataset of enhancers by using genomic windows. We collapsed the multiple cell-type annotations to unique enhancer coordinates when there are different overlapping enhancer regions, active in different cell lines, targeting the same gene and overlapping continuous windows. Our final dataset of 1,322 consensus enhancers that we used to annotate intergenic signals. When extracting the top hits under positive selection (99.9th percentile) we only took into account those enhancers that are supported by ≥ 3 databases. Similar criteria was applied when analysing the autosomal signals of positive selection in the comparison with the X.

### Luciferase Analysis

Enhancer peaks from the top candidates were selected using ENCODE signals. Ancestral (A) and derived (D) haplotypes were amplified by PCR from male (*KDM6A*: NA07357 (A), NA12003 (D); *SH2D1A*: NA18501 (D)) and female (*SH2D1A*: NA18502 (A); *HUWE1*: NA18502 (A), NA18861 (D)) individuals, after checking for homozygosity, using the following primers and the KAPA high-fidelity Taq polymerase:

KDM6A (F): 5′-CAT​CAG​AGC​TCC​TCT​AGG​CAT​GGG​AGG​GAG​T-3′
KDM6A (R): 5′-TCA​TCT​CGA​GCC​AGT​AAG​AAC​CTA​CTA​GGG​ATC​A-3′
HUWE1 (F): 5′-CAT​CAT​CTC​GAG​GAC​CAG​CCA​CTG​GGT​GTA​GT-3′
HUWE1 (R): 5′-TCA​TAA​GCT​TTA​GGG​TCC​ATG​GTC​TTC​TGG-3′
SH2D1A (F): 5′-CAT​CAT​CTC​GAG​ACA​AAT​GTT​ATT​GAT​TCC​CTC-3′
SH2D1A (R): 5′-TCA​TAA​GCT​TCG​ACC​TAA​AAG​AGT​ATA-3′

Cloning into the PGL4.10 luciferase clone was performed by using XhoI, HindIII or SacI restriction enzymes. Renilla vector was used to normalize the values as a control of transfection. Transfection into 293T cells was performed by using Lipofectamine 3,000 (Thermo Fisher, L3000001), using 100 ng of luciferase and 1 ng of Renilla control vector and maintained for 48 h in OptiMEM. Cells were harvested and luciferase activity was measured using the Dual-GLO kit (Promega, E2920). Luciferase/renilla ratios were calculated in 4 replicates and 2 independent experiments.

## Results

We inferred recent positive selection in human X chromosomes using genomic data of 1,511 individuals from 15 populations. We conducted selection scans by applying the haplotype-based statistics iHS, iHH12, and nSL, which were designed to detect signatures of hard and soft sweeps (see Methods for details) and can be used as complementary selection tools. To assess whether a region has evolved under recent positive selection, we performed coalescent simulations with *msms* (Ewing and Hermisson, 2010) to build the expected distributions under neutrality, considering human demography and the particular ascertainment bias of our data. We observed a good fit of our neutral model by comparing the observed site frequency spectrum (SFS) of the fifteen populations with neutral simulations (Supplementary Figure S1). Small deviations in singletons were observed in some populations, but with a tight fit of alleles segregating at intermediate and high frequencies.

### Regions Under Putative Positive Selection

The per-SNP metric scores might reflect the presence of particularly homozygous regions, which could indicate a selective sweep. In order to detect these signatures, selection scores were averaged separately across sliding overlapping windows (see Methods; Supplementary Figure S2), however care must be taken when interpreting these results since signatures of positive selection might expand beyond the region under positive selection. This caveat is inherently associated with window-based approaches like in this analysis, where signals exhibited by neighbouring genes might be the result of a single sweep. Most populations show distributions with larger tails compared with simulations (Supplementary Figure S2A). We considered two cut-offs based on the simulated data (99th and 99.9th) in order to extract the putatively selected windows in the empirical distributions (Supplementary Table S1).

Putative selective sweeps in regions under positive selection might present different degrees of “softness.” As noted by different authors, hard and soft sweeps are sometimes difficult to differentiate (Messer and Petrov, 2013; Schrider et al., 2015), and regions under selection might be captured by methods designed to detect both selection processes. In order to study the similarity in the regions under selection, we assessed the degree of overlap between the signals reported by the three metrics. Under the 99th percentile in the global population, the general trend shows that iHH12 presents a similar proportion of commonly targeted regions as with iHS and nSL (∼60%), while iHS found fewer common regions compared with nSL (∼36%). This could be expected since iHH12 and nSL are sensitive to both hard and soft sweeps (Ferrer-Admetlla et al., 2014; Torres et al., 2018), and iHS depends on recombination rate. The signal overlap indicates that some regions might present mixed properties of hard and soft sweeps, which could be due to the mode of selection, the degree of softness, or a linked selection effect (Schrider et al., 2015).

We observed a larger proportion of signals that fall outside the simulated distribution in the African populations in comparison with non-Africans. These results are in line with previous reports which show that the number of detectable selective sweeps by haplotype-based statistics is correlated with the effective population size (Johnson and Voight, 2018; Voight et al., 2006) (Supplementary Table S1). When comparing both hard and soft selection processes, we observed that soft-sweep-like signals reported by nSL and iHH12 are more abundant and widespread along the X chromosome, as was previously reported at genomic level (Messer and Petrov, 2013; Schrider and Kern, 2017).

The analysis reveals that high values are clustered in specific spots of the X chromosome, indicating the presence of putative selective sweeps in these regions (Figure 1) (Voight et al., 2006). The distribution of signals of selective sweeps along the X chromosome is more similar between non-African than with African populations, indicating a common clustering of extreme signals among the different out-of-Africa populations. This was noted by Pickrell et al. (2009) and might reflect the common origin of the out-of-Africa populations.

### Comparison With Autosomes

The unique inheritance rules of the X chromosome might generate different selection patterns compared with the rest of the genome. In order to contrast the X chromosome signatures, we assessed selection on the autosomes of three populations of reference (Yoruba, YRI; Utah residents with northern and western European ancestry, CEU; Han Chinese, CHB) and compared the score distributions in the three haplotype-based statistics (iHS, nSL, iHH12). We see similar patterns of selective sweeps across the different populations as in the X: a higher number of outlier regions fall into the extreme tails of the autosomes in Africans (YRI) than Europeans (CEU) or Asians (CHB) (Supplementary Table S2). As seen in the X, a higher number of windows under selection are captured by the statistics nSL and iHH12 in comparison with iHS across the autosomes, probably due to the higher presence of soft-sweep-like signatures across the genome. We evaluated the nSL scores in the top distribution quartile and decile, and found significant differences between the X chromosome and the pooled scores of autosomes (CEU, Kruskal Wallis: 36.04, *p* = 1.93e-09; CHB, Kruskal Wallis: 93.62, *p* = 3.81e-22) (Supplementary Figure S3). These higher values might be a reflection of the effect due to the haploid state in males and the smaller effective population size of the X (Veeramah et al., 2014; Johnson and Voight, 2018). However, it is difficult to associate these differences with a higher selection efficiency due to the faster-X effect, since the top 1% shows inconsistent distributions across the genome due to the presence of extreme outliers. This result might indicate that the faster-X effect is not properly captured with these selection statistics and other causes might be generating the observed differences in the distribution.

### Gene Ontology in the Candidate Regions

Generally, the closest gene to the estimated sweep is considered the best candidate for the target of selection. Putative selected regions were annotated as genic (≥1 bp overlaps with Refseq gene coordinates) and non-protein coding (Supplementary File S1). We do note that the strongest and widest signals are likely to span more than the target of selection.

To determine which processes are likely under selection, we performed a functional enrichment analysis with *Webgestalt* (Liao et al., 2019) on the top 100 genes across all populations. There is an overall enrichment in neural-related terms in the three continental groups (Supplementary Table S3A). We report synaptic and dendrite-related terms (e.“postsynapse” (GO:0098794), “dendrite” (GO:0030425)) with genes like *DMD*, *IL1RAPL1* and *GABRA3*, among others; and in general, more brain-related terms, like “nervous system process” (GO:0050877) or “cognition” (GO:0050890). Additionally, we observe genes consistently selected in continental groups which do not correspond with any enriched term. In African populations we found members of the arylsulfatase family (*ARS*) and steroid sulfatase (*STS*) gene (Holmes, 2017) under positive selection. These genes are involved in hormone metabolism, they are associated with X-linked diseases like chondrodysplasia punctata (Franco et al., 1995) and ichthyosis (Basler et al., 1992), and have a strong signal of selection (99.9th) in African populations. We also observe reproduction-related genes, like *SPANX-A1/A2/C/D* and *SPANX-OT1* in non-African populations. These genes belong to the spermatogenesis-related gene family *SPANX-A/D*. This is a highly paralogous hominin-specific group of genes which are expressed post-meiotically in testis and some cancer types (Westbrook et al., 2006) and have previously been reported as positively selected (Kouprina et al., 2004; Casto et al., 2010) and related to male fertility (Urizar-Arenaza et al., 2020). We observe signals of positive selection on the *BMP15* gene, related to ovarian insufficiency in women and subjected to positive selection in Hominidae clade (Ahmad et al., 2017). Other spermatogenesis-related genes (*SAGE1*, *SEPT6*, *CDK16*) and genes involved in human fertility (*ADGRG2, DIAPH2, FAM122C*) also appear in the highest scoring regions (99.9th) of our scans (Supplementary File S3). Following the same criteria as in the X chromosome, we performed a functional enrichment analysis on the pooled set of autosomal signals reported by the three selection statistics. As shown in Supplementary Table S3B, there is an overall enrichment in neural-related terms across selection statistics, together with processes related to kidney or smooth muscle development, as well as activities involved in endothelial growth. Similarly to the X chromosome, non-African populations (CEU and CHB) show poorer enrichment in GO terms, however there is a remarkable presence of enriched neural-related processes and other terms that involve sarcolemma and the renal system development.

### Shared Sweeps in Human Populations

Previous reports have shown that signatures of positive selection are often shared between different human populations (Johnson and Voight, 2018). Common evolutionary trajectories might generate similar selective pressures which leave shared signatures of selection. These common patterns might reveal important traits that were crucial in the adaptation of ancestral populations. To that end, we assessed the degree of overlap among candidate regions under putative positive selection. We considered the 99th percentile candidates in the three tests and identified regions whose genomic coordinates overlap across multiple populations. We observe that 41% of the selective sweeps are unique to a specific population, 38% are shared between populations of the same continental group, and 20% are shared across different continents. These results are in line with previously reported selection patterns (Johnson and Voight, 2018): common sweep events are more frequent between closely related populations, and cross-continental sweeps are rarer and more likely to result from common selective pressures and older processes of positive selection.

Among the cross-continental selected regions we find that one of the most common falls within the *DMD* (dystrophin) gene. This is the largest gene in the human genome and is involved in the stabilization of the sarcolemma and synaptic transmission. We find multiple signatures of hard and soft sweeps across the 15 populations, which together span a region which reaches up to ∼2 Mb (Supplementary Figure S4A). The variable length of this sweep might indicate that multiple selection events took place in the three continental groups, generating different patterns. Positive selection signals were previously reported in several components of the dystrophin protein complex (*DPC*) (Williamson et al., 2007) in non-African populations and in *DMD* in Africans (Casto et al., 2010). Our *DMD* results are complementary to these previous studies and validate evidence for adaptations in neurological and muscle-related phenotypes in other populations.

Another globally shared region contains the *F9* gene, which encodes the coagulation factor protein FIX and is involved in Hemophilia B. In this case, the *F9* region harbours windows under positive selection in the 99.9th percentile reported by iHH12, and reflects a region that spans up to ∼50 kb (Supplementary Figure S4B). A previous study reported coagulation factors underwent positive selection in different clades (Rallapalli et al., 2014), which might be a consequence of selective pressures due to the direct relationship with the immune system and host-pathogen interactions. Although the FIX factor has not been identified as related to any selective pressure to date, it might be under recent positive selection in human populations due to its role in the coagulation system as the first line of defence against pathogens.

### TENM1 Gene

**FIGURE 2:**
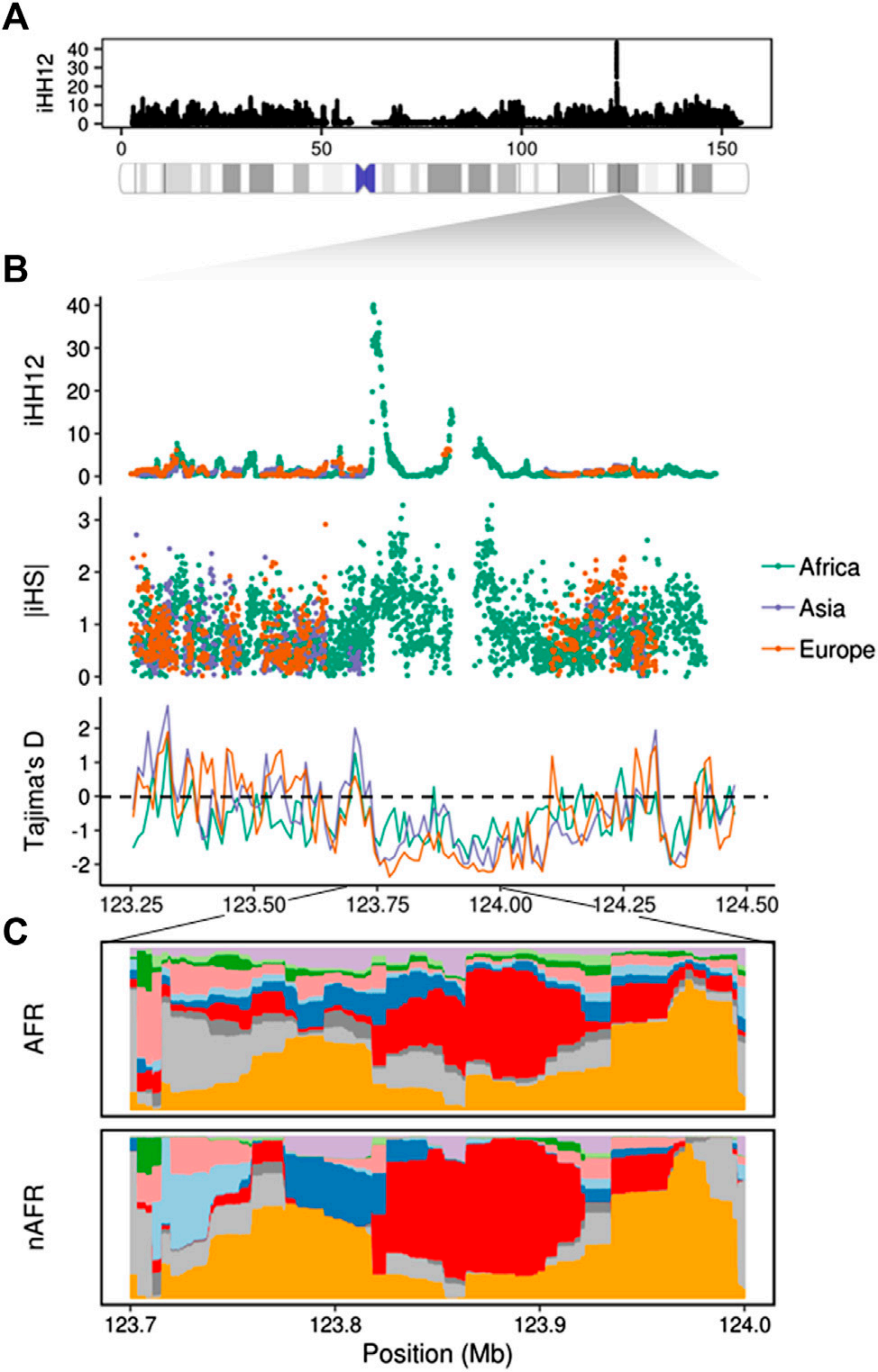
Putative positive selection signal on the *TENM1* gene. **(A)** Whole chromosome iHH12 scores in the global sample. **(B)** Manhattan plot showing the iHH12, iHS, and Tajima’s D scores on the *TENM1* gene region. **(C)** Clusters of highly similar haplotypes (in red and orange) estimated by *fastPHASE* were found in African (AFR) and non-African populations (nAFR). The different colouring represents changes in the haplotypic composition through the region, where each row represents a haplotype and each column a SNP.

The most extreme signals in the analysis are reported by iHH12, reaching values between 10 and 15 in African populations (>99.97%). Patterns of soft and incomplete hard sweeps might be a side effect of linked regions targeted by complete hard sweeps, referred to as the “soft sweep shoulder” (Schrider et al., 2015). A possible example of this is seen in the *TENM1* gene, which is the highest scoring region in the chromosome with an iHH12 signal composed of two peaks (Figures 2A,B). This gene is involved in neural development and is specifically determinant for the synapse organization of the olfactory system. In African populations this region exhibits a peak value of iHH12 > 40, and is largely filtered out by *selscan* for the non-African populations due to low minor allele frequency variants. iHS and nSL outlier windows are also found within this region, suggesting the presence of haplotype patterns which fit both soft and hard sweep signatures. In order to elucidate the haplotype structure of this region, we inferred clusters of similar haplotypes with *fastPHASE* (Scheet and Stephens, 2006) using representative populations of the three continental groups (CEU (Europe), CHB (Asia) and YRI (Africa)). Figure 2C shows different haplotypes at high frequency with two highly homozygous clusters overlapping the iHH12 peaks in African or non-African populations. This pattern is expected in regions that underwent selection processes and left long, unbroken haplotypes where no recombination events occurred. The two main clusters span ∼300 kb of the *TENM1* gene and their location suggests an ancient, strong selection event took place before the population split in the out-of-Africa event. For confirmation, we calculated the Tajima’s D statistic, which was designed to detect departures from the standard neutral model and is suited to detect ancient complete sweeps (Pybus et al., 2015). Figure 2B depicts the region with an ancient complete hard sweep with windows of Tajima’s D ≤ -2 (1% extreme). This suggests that, despite not observing iHH12 signals in non-African populations, the underlying haplotype pattern reflects a signature of positive selection among the global population. No clear phenotype could be associated with this signal, however recent evidence indicates mutations in *TENM1* are linked with congenital general anosmia (Alkelai et al., 2016), suggesting the potential for olfactory adaptations. Previous studies have shown the importance of the olfactory system in the evolution of *Homo sapiens* (Hoover, 2010) and that olfactory receptors were subjected to non-neutral selection (Hoover et al., 2015) accounting for population-specific phenotypic variability (Trimmer et al., 2019). This evidence suggests that olfactory receptors, and the associated neural system, might be subjected to important adaptive processes in human evolutionary history.

### Selection of X-Inactivation Escape Genes

The incomplete inactivation of some genes during the process of gene dosage compensation in females, might expose these escapees to sex-especific adaptive processes due to its biased expression. We wanted to investigate whether patterns of positive selection could be detected among the genes that escape X inactivation. We obtained the XCI status from Tukiainen et al. (2017) and defined 59 genes as “escape” and 381 genes as “inactive,” keeping only genes with the strongest support. We constructed contingency tables based on these categories performing Fisher’s exact test of independence between selection and XCI status for different extreme tail thresholds of the selection tests. We found that genes which escape from inactivation had a higher probability of being targeted by positive selection according to two of the tests. This trend is significant for iHS, is marginally significant for iHH12, and does not reach significance for nSL (Supplementary Tables S4A,B). Notably, escape genes under positive selection had similar proportions from iHS and iHH12 (19 and 20%, respectively; Supplementary Table S4A), however only reached 11% for nSL. This may suggest that escape genes are more likely to be targeted by selection processes that leave signatures closer to hard sweeps rather than soft sweeps.

Supplementary Table S4C lists the genes under selection that escape inactivation. On this list, we found enrichment in sulfuric ester hydrolase activity (GO:0008484), due to the sulfatase group of genes. Among these top candidates, we found four members of the *ARS* family. Three of these members participate in bone and cartilage matrix composition during development (*ARSE*, *ARSD*, *ARSF*). These genes are associated with the X-linked Chondrodysplasia Punctata, a syndrome that affects almost exclusively females, and is characterized by abnormal embryo development, including skeletal malformations, skin abnormalities, and cataracts (Franco et al., 1995).

The *STS* gene, also escaping inactivation, presents another shared region among populations (iHS 99.9^th^ percentile in African populations and 99th percentile in Europeans and Asians). It is associated with X-linked Ichthyosis, a syndrome caused by a placental steroid hormone deficiency and is characterized by skin and eye abnormalities (Basler et al., 1992). This gene was reported to be one of the top female-biased genes differentially escaping inactivation in Yoruba (YRI) (Johnston et al., 2008). As hypothesized by Tukiainen et al. (2017), most of the escape genes reported as under selection show female-biased expression, suggesting these genes might be involved in some sex-based adaptive trait.

### Functional Non-coding Regions Under Positive Selection

Previous studies have reported numerous signatures of positive selection with an unknown genic cause. This might be accounted for by a high false positive rate in genomic scans but also by the presence of signatures in non-genic regions, suggesting that many true signals are located in non-coding, potentially regulatory elements (Fraser, 2013; Enard et al., 2014).

In order to identify the strongest and most interesting candidates of positive selection, we evaluated the signals in the 99.9th percentile and attempted to pinpoint the target of selection within each signal by annotating SNPs with *ANNOVAR* (Wang et al., 2010). A large portion of SNPs over the 1% per-SNP score extreme tail are intergenic and a large fraction fall within intronic regions for all statistics (iHS: 29%, iHH12: 32%, nSL: 20%), with few in exons or untranslated regions. CADD scores (Kircher et al., 2014) were used to identify functional variants according to their deleteriousness (see Methods). After filtering by functionality (CADD ≥10), the majority of the variants were excluded, however, the SNP composition remained higher in intergenic regions (Supplementary Table S5), with an average prevalence in signals reported by iHH12 and nSL in non-African populations (Africa: ∼62%, Europe: ∼72%, Asia: ∼90%). These results suggest that there is an excess of signals driven by intergenic SNPs that fall in non-annotated and potentially regulatory regions.

Several intergenic regions are under positive selection in the different continental groups (Supplementary Table S1). In order to assess the functional impact of these signals, we explored the overlap of the extreme SNPs within the 99.9th percentile windows with RegulomeDB (Boyle et al., 2012) annotated elements. The combined signals across all populations had higher proportions of SNPs within an ENCODE element (iHS: 19.1%, iHH12: 26.3%, nSL: 13%) compared to the whole chromosome (5.5%). This enrichment is more prevalent for iHH12 signals, which may be due to its power to detect both hard and soft signatures. This finding shows, as expected, intergenic regions under putative positive selection are enriched in functional elements and likely points to selection of regulatory processes.

Intergenic signals cluster around genic regions, suggesting a regulatory function influencing surrounding genes. Under the 99th percentile, we found instances of genic windows that present a partial overlap with genes, presenting both genic and intergenic SNPs. The presence of these partially overlapping windows is more prevalent among the candidate regions under positive selection reported by iHH12 and nSL statistics (iHS: 2%, iHH12: 5.7%, nSL: 4.4%) across all populations. Since regulatory elements are expected to be found in the extremes and within coding regions, we used the RegulomeDB annotation to associate the signal of putative selection with any potential regulatory function. In these overlapping regions we found that the odds of intergenic SNPs overlapping a functional element is higher than genic SNPs (Supplementary Table S6A) according to iHH12 and nSL, moreover, when considering extreme SNPs (99.9th), these values reflected a much higher dominance of functional intergenic SNPs (Supplementary Table S6B). These findings indicate that the overlapping genic windows under selection are more enriched in regulatory elements in their intergenic portion, something that points to the presence of sweeps in regulatory elements.

This evidence suggests, as previously noted, amino acid changes may play a less important role in recent adaptation and regulatory changes may drive a more important part of adaptation events in recent human evolution (Fraser, 2013; Grossman et al., 2013; Enard et al., 2014).

### Enhancer-like Signatures Under Positive Selection

In order to analyze in more detail the regulatory roles of the regions under putative positive selection, we intersected the intergenic windows in the extreme tails with the enhancer coordinates described in the HACER database (Wang et al., 2019). Supplementary Table S7A shows the overlapping/non-overlapping windows with enhancer regions (in any cell line) in the 99th percentile extreme tail. We find that selection is more probable in intergenic regions with overlapping enhancers. A similar pattern of enrichment to the sexual chromosome is seen in the autosomes. iHH12 presents higher proportions of overlapping windows than iHS and nSL, suggesting that enhancers are enriched among the captured autosomal signatures of positive selection (Supplementary Table S7B). Moreover, unlike in the X chromosome, this enrichment is more pronounced in non-African populations, suggesting that processes of regulatory adaptations might have been incremented since the Out of Africa event.

In the X chromosome, several of these enhancers were located close to genes also reported as positively selected in the analysis. We wanted to determine if this pattern is a by-product of the selection in adjacent regions by genetic linkage (hitchhiking effect), or due to independent selection processes on both elements. To account for the cell-type-specific enhancers described in HACER, we created a consensus enhancer dataset (see Methods) with unique coordinates. We pooled all populations and selection tests in order to maximize the statistical power of our analysis. A Chi-squared test shows the association between selection of enhancers and their target genes (*p*-value = 0.0021). However, despite the association between these two variables, we observe a higher probability that both elements are under positive selection (YY category) than expected by chance (Supplementary Tables S8A,B). We compared the mean distance between the selected/non-selected enhancers and their closest selected/non-selected genes. These distances do not support the physical genetic linkage as a possible explanation of the association. It must be taken into account that the reported distances are sometimes too large (∼2.5 Mb) to be the reason for selection by hitchhiking of both elements. Therefore, the YY set of enhancers and target genes must be regions that are jointly swept by hitchhiking and few regions selected by independent processes. This suggests that selective pressures might affect some genes and their regulatory elements in a coordinated way, modifying not only their coding sequence but also their expression level.

Next, we wanted to study the potential origin of some of the most extreme intergenic signals and the regulatory effect of the sweeping haplotypes. We focused on the highest scoring candidate enhancers (99.9th) and their closest genes (Supplementary Table S9). Among candidates at X:73,135,561-73,145,161, an African-shared signal (iHS) that overlaps an enhancer (Supplementary Figure S6) located in the XIC region (X-inactivation center) and whose closest gene is *JPX*. This region is active in five different cell lines according to HACER (H1, HUVEC, HCT116, AC16, REH) and is supported by three databases (Ensembl Regulatory Build, ENCODE Enhancer-like Regions and ChromHMM). The gene *JPX* (∼23 kb away) is an activator of the lncRNA *XIST*, which is involved in the X chromosome inactivation. Among the potential causal variants, the SNP rs112977454 reported as eQTL by the GTEx project, is the most likely candidate. In addition, this eQTL has a CADD score of 9.018, close to the 1% pathogenicity threshold (CADD = 10) used by Kircher et al. (2014), and an average derived allele frequency (DAF) of 17% in African populations, and is absent from the rest of populations. This eQTL overlaps a TFBS in the HUVEC cell line, which targets the *JPX* gene through the transcription factors *FOS*, *GATA2*, *JUN*, and *POLR2A*. No specific phenotype is associated with this variant; however, these results suggest that it might influence the transcription factor binding and affect the regulation of the *JPX* gene.

### Functional Analysis of Enhancers Under Positive Selection

**FIGURE 3:**
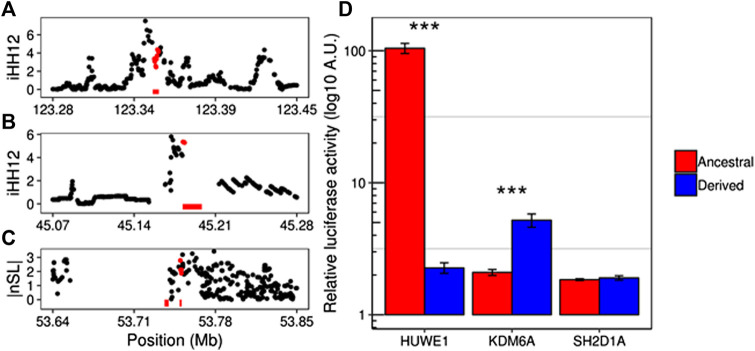
Candidate enhancers under putative positive selection. Manhattan plots show the selection scores overlapping the enhancer coordinates (bottom red bars) targeting *SH2D1A* **(A)**, *KDM6A* **(B)**, and *HUWE1* **(C)** genes in YRI, CEU, and YRI populations, respectively. Although *HUWE1* appears under positive selection in Gambians (GWD) (Supplementary Table S9), YRI individuals were used in the luciferase assay instead since the signal is also present in this population (99th percentile). Red dots correspond to enhancer overlapping SNPs. **(D)** Relative luciferase activity comparisons between the ancestral and derived haplotypes in each of the candidate enhancers. Significant differential activities are seen in *HUWE1* (*p* = 5.75e10^−8^) and *KDM6A* (*p* = 0.004) enhancers.

In order to explore the potential regulatory effect behind the selection processes in the candidate enhancers (Supplementary Table S9), we compared the regulatory activity of the putative haplotype under selection with that of its ancestral sequence. We analyzed the changes in the expression of the reporter gene luciferase under regulation of the two ancestral and derived haplotypes in *HUWE1*, *KDM6A* and *SH2D1A* (Figures 3A–C). This method allows us to test all the potential causal variants independently. All tested genes also harbor signals of positive selection in their sequences. These genes are implicated in intellectual dissability (*HUWE1*) (Giles and Grill, 2020) and the Duncan disease (*SH2D1A*) (Sumegi et al., 2002), and, in the case of *KDM6A*, this gene is reported as X-inactivation escapee by Tukiainen et al. (2017), which makes it susceptible to sex-specific processes (Dunford et al., 2017; Itoh et al., 2019). In all these cases, the enhancer region overlaps with more than one potential causal SNP. Ancestral and derived haplotypes of the candidate enhancers were obtained from males of the relevant population under selection and subsequently cloned in a luciferase-reporter vector. Upon transfection in 293T cells, significantly different luciferase activity amongst the ancestral and derived haplotypes for *HUWE1* and *KDM6A* enhancers were observed, showing a clear distinction of the regulatory activity between these two haplotypes (Figure 3D). Yet this analysis did not show differential activity between the ancestral and derived form of the *SH2D1A* enhancer. Although no specific phenotypes were able to be assigned to the selection of these regions, our data suggest that positive selection has contributed to adaptation of human populations by differentially regulating gene expression. Further studies will be needed to understand the phenotypic consequences of such adaptations.

## Discussion

In this analysis, we report a comprehensive analysis of recent positive selection in the X chromosome of 15 non-admixed sub-Saharan African, European, and East Asian populations. We focused on signatures recognized by the selection statistics iHS, iHH12, and nSL, which are based on the detection of extended long haplotypes at moderately high and intermediate frequencies (hard and soft sweeps). These three statistics have differing approaches and statistical power to detect the different modes of selective sweeps. However, in some cases, the similarity of the haplotypic patterns behind hard and soft sweep signals might lead to simultaneous detection of the same selected region by these methods. Results indicate Sub-Saharan African populations have a higher proportion of windows under selection than Europeans and Asians. This is directly related to the effect of haplotype-based statistics, in which the number of detectable windows under selection is correlated with the effective population size (Voight et al., 2006; Johnson and Voight, 2018). In contrast with iHS, a higher number of soft sweep-like signatures is presumably captured by nSL and iHH12 statistics. This was previously noted by authors who claimed that regions targeted by hard sweeps are much less common than soft sweeps (Messer and Petrov, 2013; Schrider and Kern, 2017). Subtle changes of frequency in multiple loci might be responsible for numerous quantitative adaptations that would require a more profound and comprehensive analysis than the one conferred by the “sweep” vision (Höllinger et al., 2019). Therefore, it is more likely that genomes, and the X chromosome, are populated by a greater number of signatures with different degrees of “softness” which are misclassified or overlooked by most selection statistics.

The faster-X effect is believed to act on the X chromosome when the hemizygous state leads to a complete penetrance of mutations, allowing for a quicker and stronger adaptive process. Differences between autosomes and the X chromosome are observed for the nSL statistic among non-African populations, and may suggest some kind of effect that generates the skewed distributions. However, these differences could not easily be associated with the faster-X effect due to the lack of a clear pattern in the top 1%. However, as previously noted by Arbiza et al. (2014), natural selection seems to be a more powerful force in the sexual chromosome than in autosomes, which might explain differences in X/autosome diversity in human populations. Particular selection events and sex-biased processes might leave specific pronounced signatures in the X chromosome, as we report in this paper. Nevertheless, despite accounting for demography and different mutation rates in our simulations, selection is not the only factor that could be invoked to explain the differences in haplotype diversity. It is clear that the selection of genes involved in neural development is ubiquitous and similar between the X and the autosomes, reflecting comparable patterns between different populations.

We report signals of recent positive selection in particular regions of the X chromosome. The difficulty of identifying clear signals from particular selection processes relies on the mixed properties of most signatures. In our scan most of the observed signals are captured by more than one statistic. One of the most remarkable cases of selection in our analysis is the *TENM1* gene. This gene harbours ∼300 kb region (Figure 2) with selection signals that indicate an old and strong event of positive selection before the populations split. Moreover, this region matches with a recombination hotspot, which might have affected the underlying haplotype pattern. The haplotype clusters inferred by *fastPHASE* show a clear predominance of two types of sequences that could derive from a unique sweeping haplotype which could be broken by recombination. Although the role of *TENM1* selection might be linked to recent changes of the olfactory system, the origin of the haplotype patterns seen in our analysis could have more general implications for neural development. Genic regions under putative positive selection seem to be dominated by genes involved in neural-related processes. This is widely reported by the tests used and appears to be globally distributed. These findings fit with previous evolutionary studies which describe the role of neural genes in recent human history (Wei et al., 2019).

Commonalities with previous studies reinforce evidence of X-linked selection in human populations. We found complementary results such as selection in *DMD* or reproduction-related genes like the *SPANX* family, and expanded the findings in new populations and genes. It is of interest to remark on the case of the *SPANX* members and other reproduction-related genes. The potential importance of fertility-related genes in recent human history was previously reported (Ramm et al., 2014; Hart et al., 2018). The *SPANX* members, as well as other cancer-testis genes like some melanoma antigen gene (*MAGE*) family members detected in our analysis, are known to be under rapid evolution and appear to be subjected to positive selection affecting their coding sequences (Kouprina et al., 2004). Previous reports found members of the spermatogenesis-related family *SPATA* to be under recent positive selection and suggest that testis-enriched genes are the target of population-specific selection (Schrider and Kern, 2017; Schaschl and Wallner, 2020). Other studies report specific ampliconic gene-enriched regions in humans and primates were targeted by strong selective sweeps, where meiotic drive and sperm competition seem to be a potential explanation (Dutheil et al., 2015; Nam et al., 2015). Although an important number of previously reported genes under selection have been captured in our scan, it is important to note that a high FDR is expected from this “hypothesis free” approach. Nonetheless, despite the likely presence of false positives, our findings are in line with previous evidence and supports the importance of reproduction and male fertility in recent human evolutionary history.

Dosage compensation of X chromosome genes occurs in females by the random inactivation of one of the copies during the early stages of embryogenesis. However, this process of transcriptional silencing is not complete for all the genes. Evidence suggests that ∼23% of the X-linked genes “escape” inactivation and both chromosomal copies are expressed. Most of these genes are located in the PAR1 and only a small fraction is distributed in the nPAR (Balaton et al., 2015; Tukiainen et al., 2017). Overall, our analysis shows an enrichment of genes under selection which escape X-inactivation mainly driven by hard sweeps. These genes were previously described as likely being under purifying selection (Park et al., 2010), however, no evidence for positive selection has been reported until now. It is possible that background selection might produce false positives for haplotype-based statistics, however a recent report has shown that this kind of selection is not likely to mimic the signatures of selective sweeps (Schrider, 2020). Although background selection has not been tested, the haplotype-based statistics used in this analysis are not expected to be affected by this kind of selection. Therefore, these X-linked escape genes which have biased expression between sexes and might be responsible for sexual dimorphic traits, likely producing phenotypic diversity which has been adaptive during human evolution. Additional analyses on escape genes are needed in order to establish a phenotypic cause for such adaptations.

A large fraction of regions under selection has no annotations. We report evidence of intergenic regions with high selection scores, reflecting the presence of signatures which fit the two selection processes we consider in this analysis. Enrichment in the regulatory elements annotated by RegulomeDB is seen globally in the two selection processes, with a higher prevalence in regions exhibiting soft sweep-like signatures (iHH12 and nSL signals). Sometimes genic regions might be affected by the selection of the surrounding intergenic regions with regulatory elements. In our analysis we found cases of selected windows with genic and intergenic portions (iHS: 2%, iHH12: 5.7%, nSL: 4.4%). In the intergenic portions, these windows exhibit an enrichment of highly scored SNPs overlapping functional elements annotated by RegulomeDB, suggesting that selection is driven by regulatory elements.

A recent analysis of selection in enhancers revealed that approximately 5.90% of the enhancers studied in different tissues present signatures compatible with recent positive selection events (Moon et al., 2019). Other evidence of selection in enhancers has demonstrated how a SNP subjected to positive selection is able to modify the regulatory activity of the region in a population specific manner (Nakayama et al., 2017). With this in mind, we used the HACER database to study the potential role of selection in active human enhancers. We show several cases of reported enhancers under selection whose closest gene (the assumed target) is under putative positive selection in our analysis. This result might reflect a linkage effect between these two elements; however, we suggest that in some cases this is an indication of concurrent selection of the gene and regulatory region. We also report that not only in the X chromosome, but also in the autosomes, enhancers are more likely to be present in regions under putative positive selection. Both African and non-African populations seem to present a significant contribution of regulatory elements to the origin of selection signatures, and therefore participate in processes or regulatory adaptations. We report specific cases of putative positive selection signals in enhancers which might drive population-specific regulatory changes. African populations had a high scoring hard sweep-like signature in an enhancer located in the XIC region. Among the top SNPs, we find rs112977454 (99.96th percentile) as an eQTL segregating in Africans which might affect the binding of transcription factors that regulate the expression of the lncRNA *JPX*. This gene is a key participant in the X chromosome inactivation as it promotes the expression of *XIST* (Tian et al., 2010), which silences transcription by coating the chromosome into the Barr body. This is an interesting candidate since it might affect expression patterns of genes that escape from the X-inactivation and thus plays a role in the potential adaptations of dimorphic traits. Although this SNP seems to be the most likely cause of the selection signature, the detection of the causal mutation is an extremely difficult task and further analyzes would be needed to pinpoint the driver allele.

In order to reveal the potential regulatory effect of our enhancers under selection, we performed luciferase-based assays on three of our top candidates. *HUWE1* and *KDM6A* enhancers exhibit a significant difference in the luciferase activity between the two most differentiated haplotypes. This effect clearly suggests a differential regulation of these genes which might fit with the hypothesis of population-specific selection processes. The case of *KDM6A* is rather remarkable since it has been associated with female-specific traits where its ability to escape from the X-inactivation plays a significant role. The biallelic expression of this gene seems to confer a protective effect in females in a wide range of cancer types, in which males are more exposed due to their hemizygous state (Dunford et al., 2017). The same overexpression of *KDM6A* appears to be involved with sex differences in autoimmune disease susceptibility, contributing to a higher incidence of multiple sclerosis in females (Itoh et al., 2019). Although we were not able to make a direct association between our selection signals and these phenotypes, the effect of selection in these enhancers and the potential role of adaptations in escape genes suggest that selection might be affecting sex-specific secondary processes. Like in other genomic scans, the strategy to detect selection followed in our analysis might be limited and miss certain regions or modes of selection contributing to the landscape of positive selection in the human X chromosome. This, together with the inherent difficulty of identifying the precise target of natural selection, make this type of analysis a challenging aspect in the study of evolution.

## Data Availability

The original contributions presented in the study are included in the article/Supplementary Material, further inquiries can be directed to the corresponding author.

## Footnotes

1 R Core Team (2019). R: A Language and Environment for Statistical Computing. *R. Found. Stat. Comput*. Available at: https://www.r-project.org/.

## Abbreviations

CADD, combined annotation dependent depletion; DAF, derived allele frequency; EHH, extended haplotype homozygosity; eQTL, expression quantitative trait loci; GO, gene ontology; HACER, human active enhancer to interpret regulatory variants database; HMM, hidden markov model; iHH12, integrated haplotype homozygosity pooled test; iHS, integrated haplotype score test; Kb, kilobases; LD, linkage disequilibrium; MAF, minor allele Frequency; Mb, megabases; nPAR, non-pseudoautosomal region; nSL, number of segregating sites by length test; OEA, overrepresentation enrichment analysis; PAR, pseudoautosomal region; SFS, site frequency spectrum; SNP, single nucleotide polymorphism; TFBS, transcription factor binding site; XCI, X chromosome inactivation; XIC, X-inactivation center.
