# Supplementary material for: Chromosome X-wide Analysis of Positive Selection in Human Populations: Common and Private Signals of Selection and its Impact on Inactivated Genes and Enhancers
Source: Front Genet. 2021 Sep 27;12:714491. doi: 10.3389/fgene.2021.714491 (PMC8502928; doi:10.3389/fgene.2021.714491)
Supplement: Supplementary file 1 [file DataSheet2.PDF]

Group	Population	iHS		nSL		iHH12	
		99th	99.9th	99th	99.9th	99th	99.9th
AFR	ESN	142	25	185	39	172	51
	GWD	128	32	180	42	166	46
	MSL	153	40	188	39	168	51
	LWK	173	28	190	45	171	59
	YRI	143	31	172	35	181	53
EUR	CEU	61	12	23	0	84	9
	FIN	64	13	29	1	70	15
	GBR	83	13	19	4	88	12
	IBS	58	7	28	8	83	13
	TSI	36	8	21	1	81	12
ASI	CDX	53	9	29	4	57	19
	CHB	53	6	32	3	67	11
	CHS	57	4	28	6	55	12
	JPT	55	7	38	1	54	16
	KHV	56	6	31	4	60	14

Supplementary Table 1. Windows under putative positive selection in the extreme simulated 99th and 99.9^{th} percentiles across the 15 populations under study and the three selection statistics accounting for hard and soft sweeps (iHS, iHH12 and nSL).

	iHS			nSL			iHH12		
Chr	YRI	CEU	CHB	YRI	CEU	CHB	YRI	CEU	CHB
1	180	81	74	229	51	23	277	108	96
2	191	98	118	325	51	41	273	130	122
3	228	46	99	205	33	35	224	101	87
4	193	88	83	275	59	29	207	91	98
5	146	68	72	248	52	16	205	110	95
6	119	60	48	230	35	23	239	114	98
7	123	53	55	190	51	24	180	84	70
8	120	39	51	202	38	26	178	81	62
9	69	41	71	161	32	25	132	52	68
10	123	48	54	156	42	19	145	72	70
11	120	45	45	169	42	30	165	56	68
12	86	32	60	196	24	14	170	62	53
13	72	35	34	130	32	19	124	65	53
14	79	34	34	117	30	18	109	50	39
15	66	18	28	85	16	8	66	35	27
16	61	16	30	95	17	8	87	42	36
17	50	27	28	63	17	7	71	40	22
18	65	13	13	95	23	6	92	36	30
19	31	9	9	73	5	6	54	18	19
20	41	9	21	67	6	6	65	26	24
21	31	11	14	45	6	7	42	18	23
22	27	5	6	33	7	6	34	11	12

Supplementary Table 2. Windows under putative positive selection in the extreme simulated 99th percentiles in the human autosomes of the three populations of reference (YRI, CEU and CHB) across the three selection statistics accounting for hard and soft sweeps (iHS, iHH12 and nSL).

Group	Population	Test	GO term	Description	FDR
Africa	ESN	-	-	-	-
	GWD	iHS	CC:GO:0005788	endoplasmic reticulum lumen	0.00056922
		iHH12	BP:GO:0050877	nervous system process	0.028361
		iHH12	CC:GO:0098590	plasma membrane region	0.036292
		iHH12	CC:GO:0042995	cell projection	0.036292
		iHH12	CC:GO:0120025	plasma membrane bounded cell projection	0.036292
		nSL	BP:GO:0007155	cell adhesion	0.014150
		nSL	BP:GO:0022610	biological adhesion	0.014150
	MSL	iHH12	CC:GO:0098590	plasma membrane region	0.044849
	LWK	-	-	-	-
	YRI	iHS	CC:GO:0043005	neuron projection	0.020794
		iHS	CC:GO:0097458	neuron part	0.020794
		nSL	BP:GO:0007155	cell adhesion	0.044903
Europe	CEU	nSL	CC:GO:0097458	neuron part	0.019036
	FIN		-	-	-
	GBR	iHS	CC:GO:0030425	dendrite	0.032376
		iHS	CC:GO:0097447	dendritic tree	0.032376
		iHS	CC:GO:0043005	neuron projection	0.030070
		nSL	CC:GO:0097458	neuron part	0.043837
	IBS	iHH12	CC:GO:1902495	transmembrane transporter complex	0.026731
		iHH12	CC:GO:0030425	dendrite	0.013228
		iHH12	CC:GO:0098794	postsynapse	0.013228
	TSI	-	-	-	-
Asia	CDX	-	-	-	-
	CHB	iHH12	BP:GO:0050890	cognition	0.041132
	CHS	-	-	-	-
	JPT	-	-	-	-
	KHV	-	-	-	-

Supplementary Table 3A. Significant GO terms of the top 100 genes across all the Sub-saharan African, European and Asian populations in the three selection tests used in the analysis. We consider FDR < 0.05 as significant. In the table, we present the population ID, the tests where the term is reported as significant, the GO term ID, the term description and the corrected FDR value.

Population	Test	GO term	Description	FDR
YRI	iHS	CC:GO:1990391	DNA repair complex	0.0087086
	iHS	CC:GO:0014069	postsynaptic density	0.0094258
	iHS	CC:GO:1990234	transferase complex	0.00022095
	iHS	MF:GO:0019208	phosphatase regulator activity	0.027141
	iHS	MF:GO:0005524	ATP binding	0.0065002
	iHS	MF:GO:0035639	purine ribonucleoside triphosphate binding	0.0065002
	iHH12	BP:GO:1901722	regulation of cell proliferation involved in kidney development	0.030640
	iHH12	BP:GO:0014910	regulation of smooth muscle cell migration	0.026302
	iHH12	BP:GO:0001764	neuron migration	0.00072455
	iHH12	CC:GO:0098890	extrinsic component of postsynaptic membrane	0.020658
	iHH12	CC:GO:0008328	ionotropic glutamate receptor complex	0.0051770
	iHH12	CC:GO:0098839	postsynaptic density membrane	0.00039174
	iHH12	MF:GO:0005021	vascular endothelial growth factor-activated receptor activity	0.045921
	iHH12	MF:GO:0017081	chloride channel regulator activity	0.047474
	iHH12	MF:GO:0008066	glutamate receptor activity	0.047454
	nSL	BP:GO:0099537	trans-synaptic signaling	0.041931
	nSL	BP:GO:0007268	chemical synaptic transmission	0.041931
	nSL	BP:GO:0098916	anterograde trans-synaptic signaling	0.041931
	nSL	CC:GO:0008328	ionotropic glutamate receptor complex	0.0057804
	nSL	CC:GO:0043197	dendritic spine	0.0015811
	nSL	CC:GO:0099146	intrinsic component of postsynaptic density membrane	0.0067098
	nSL	MF:GO:0008066	glutamate receptor activity	0.021565
	nSL	MF:GO:0099094	ligand-gated cation channel activity	0.020551
	nSL	MF:GO:0004725	protein tyrosine phosphatase activity	0.025421
CEU	iHS	CC:GO:0042383	sarcolemma	0.011196
	iHH12	BP:GO:0072001	renal system development	0.015775
	iHH12	BP:GO:0007268	chemical synaptic transmission	0.015775
	iHH12	BP:GO:0098916	anterograde trans-synaptic signaling	0.015775
	iHH12	CC:GO:0071141	SMAD protein complex	0.046792
	iHH12	CC:GO:0008328	ionotropic glutamate receptor complex	0.0073241
	iHH12	CC:GO:0099634	postsynaptic specialization membrane	0.0041002
	iHH12	MF:GO:0005261	cation channel activity	0.036554
	iHH12	MF:GO:0046873	metal ion transmembrane transporter activity	0.025939
	iHH12	MF:GO:0022890	inorganic cation transmembrane transporter activity	0.036554
	nSL	-	-	-
CHB	iHS	CC:GO:0045202	synapse	0.043032
	iHS	CC:GO:0097458	neuron part	0.013393
	iHH12	CC:GO:0031088	platelet dense granule membrane	0.043596
	iHH12	CC:GO:0043235	receptor complex	0.00011943
	iHH12	CC:GO:0045202	synapse	0.00011943
	nSL	CC:GO:0042734	presynaptic membrane	0.033748
	nSL	CC:GO:0097060	synaptic membrane	0.033748
	nSL	CC:GO:0097458	neuron part	0.033748

Supplementary Table 3B. Functional enrichment analysis on the pooled autosomal signatures of positive selection reported by iHS, iHH12 and nSL. The three populations of reference YRI (Africa), CEU (Europe) and CHB (Asia) were used as a proxy for the three main geographic groups.

		iHS			iHH12			nSL		
		Selected	Not Sel.	Total	Selected	Not Sel.	Total	Selected	Not Sel.	Total
95th	Escape	31	28	59	17	42	59	18	41	59
	Inactive	133	248	381	68	313	381	136	245	381
	Total	164	276	440	85	355	440	154	286	440
99th	Escape	18	41	59	10	49	59	9	50	59
	Inactive	44	337	381	34	347	381	50	331	381
	Total	62	378	440	44	396	440	59	381	440
99.9th	Escape	8	51	59	3	56	59	4	55	59
	Inactive	14	367	381	10	371	381	12	369	381
	Total	22	418	440	13	427	440	16	424	440

Supplementary Table 4A. Contingency tables of escape genes under selection reported by the three selection statistics across three extreme percentiles (95th, 99th and 99.9th). Two categories were used: escape/inactive and selected/non-selected.

	iHS			iHH12			nSL		
	Fisher's p	O.R	C.I.(0.95)	Fisher's p	O.R.	C.I.(0.95)	Fisher's p	O.R.	C.I.(0.95)
95th	0.01	2.06	1.14-3.73	0.05	1.86	0.93-3.57	0.46	0.79	0.41-1.47
99th	0.0003	3.35	1.66-6.59	0.06	2.07	0.86-4.64	0.68	1.19	0.48-2.64
99.9th	0.004	4.09	1.41-11.07	0.40	1.98	0.34-8.02	0.24	2.23	0.50-7.70

Supplementary Table 4B. Fisher's tests applied to the contingency tables. iHS reports significant p-values across the three extreme percentiles with increasing odds ratios (OR). iHH12 and nSL do not show significant enrichment in escape genes, however the odds are in line with those in iHS in five out of the six comparisons, suggesting the presence of selection but with lack of significance probably due to a sample effect.

	AFR	EUR	ASI
95th	AP1S2, ARSD, ARSE, ARSF, ARSH, CDK16, FAM9C, HS6ST2, HTR2C, JPX, KDM6A, MAGEC3, MAOA, MED14, MSL3, MXRA5, NR0B1, OFD1, PCDH19, PNPLA4, PRKX, STS, TMEM27, UBA1, ZCCHC16, ZRSR2	ARSF, GYG2, HS6ST2, HTR2C, MAGEC3, STS, TAF7L, TMEM27, USP9X, ZCCHC16, ZFX	FUNDC1, KDM6A, STS, USP9X
99th	ARSE, ARSF, ARSH, CDK16, FAM9C, HS6ST2, HTR2C, KDM6A, MAGEC3, MAGEC3, MED14, MXRA5, OFD1, STS, TMEM27, UBA1,	HS6ST2, STS, USP9X, ZCCHC16	FUNDC1,KDM6A, STS
99th	ARSF, ARSH, CDK16, HTR2C, KDM6A, STS, UBA1	ZCCHC16	-

Supplementary Table 4C. Escape genes reported by iHS as being under positive selection in each continental group across the extreme percentiles.

Group	Population	iHS				iHH12				nSL			
		Int	I	E	D	Int	I	E	D	Int	I	E	D
AFR	ESN	18	5	0	0	30	16	1	0	17	6	0	0
	GWD	18	11	1	0	34	30	2	0	19	9	0	1
	MSL	18	19	0	0	39	34	3	0	17	9	0	0
	LWK	32	15	0	0	56	35	2	0	18	10	0	0
	YRI	14	10	1	0	38	18	1	0	18	7	0	0
EUR	CEU	3	1	0	0	12	3	0	0	0	0	0	0
	FIN	1	4	1	0	11	6	0	0	0	0	0	0
	GBR	3	1	0	0	19	2	0	0	4	1	0	0
	IBS	2	3	0	0	9	7	0	0	5	1	0	0
	TSI	3	5	0	0	6	7	0	0	0	1	0	0
ASI	CDX	5	1	0	0	17	4	0	0	1	1	0	0
	CHB	1	0	0	0	15	0	0	0	4	0	0	0
	CHS	4	0	0	0	16	0	0	0	4	0	0	0
	JPT	1	0	0	0	16	1	0	0	0	0	0	0
	KHV	3	2	0	0	17	3	0	0	0	0	0	0

Supplementary Table 5. SNPs with a selection score within the 1% extreme and with CADD score ≥ 10 in the 99.9th percentile across all populations (Intergenic (Int), Intronic (I), Exonic (E), Downstream (D)

Test	Region	n SNPs	RegulomeDB scores					ENCODE elements	OR
			2	3	4	5	6		
iHS	Intergenic	31 (0.1)	-	-	1	1	9	35%	1.08
	Genic	286 (0.9)	-	-	6	16	74	33%	
iHH12	Intergenic	35 (0.14)	-	-	1	6	11	51%	1.32
	Genic	212 (0.85)	2	2	8	25	57	44%	
nSL	Intergenic	32 (0.12)	-	-	-	3	10	40%	1.23
	Genic	230 (0.88)	1	4	4	9	64	35%	

Supplementary Table 6A. RegulomeDB annotation of the 99th percentile genic windows with intergenic overlap and the odds ratio (OR) between genic and intergenic SNPs within functional elements. All populations are considered.

Test	Region	n SNPs	RegulomeDB scores					ENCODE elements	OR
			2	3	4	5	6		
iHS	Intergenic	4 (0.05)	-	-	-	-	1	25%	0.77
	Genic	83 (0.95)	-	-	3	3	19	30%	
iHH12	Intergenic	8 (0.08)	-	-	-	2	5	87%	8.24
	Genic	98 (0.92)	1	1	2	15	26	45%	
nSL	Intergenic	10 (0.16)	-	-	-	1	5	60%	2.11
	Genic	41 (0.84)	1	-	2	1	13	41%	

Supplementary Table 6B. RegulomeDB annotation of the 99th percentile genic windows with intergenic overlap when considering extreme scoring SNPs (per-SNP 1% extreme tail). iHH12 and nSL show a significant OR increment in comparison with iHS

Test	Group	99th				99.9th			
		Non-ovlp	Ovlp.	%	OR	Non-ovlp	Ovlp.	%	OR
iHS	AFR	194	3	1.52	0.46	47	3	6.00	1.93
	EUR	116	1	0.86	0.26	23	0	0	0
	ASI	111	7	5.93	1.93	17	0	0	0
iHH12	AFR	128	17	11.72	4.31	43	3	6.52	2.11
	EUR	77	4	4.94	1.57	17	2	10.52	3.57
	ASI	79	8	9.20	3.14	22	1	4.35	1.37
nSL	AFR	283	4	1.39	0.41	68	2	2.86	0.88
	EUR	52	1	1.89	0.58	8	1	11.11	3.77
	ASI	77	2	2.53	0.78	8	0	0	0

Supplementary Table 7A. Overlapping and non-overlapping intergenic windows under putative positive selection on enhancer regions reported by HACER in any cell line (see Methods) across the three continental groups. Odds ratio (OR) of intergenic and overlapping windows shows a significant increment mainly in iHH12 across all populations.

Test	Group	99th			
		Non-ovlp	Ovlp.	%	OR
iHS	Africa	3794	225	5.93	0.907
	Europe	1569	142	9.05	1.396
	Asia	2116	178	8.41	1.298
iHH12	Africa	5176	488	9.43	1.480
	Europe	2824	357	12.64	1.99
	Asia	2434	287	11.79	1.844
nSL	Africa	4269	263	6.16	0.943
	Europe	1102	76	6.90	1.058
	Asia	646	41	6.35	0.973

Supplementary Table 7B. Overlapping and non-overlapping intergenic windows under putative positive selection on enhancer regions reported by HACER in any cell line (see Methods). We used the pooled set of windows reported by the three tests (iHS, iHH12 and nSL) in the three populations of reference.

		Observed selection on target gene		
		Y	N	Total
Observed selection on enhancer	Y	218	350	568
	N	167	395	562
	Total	385	745	1130

		Expected selection on target gene		
		Y	N	Total
Expected selection on enhancer	Y	193	375	568
	N	192	370	562
	Total	385	745	1130

Supplementary Table 8. Contingency tables of both observed and expected pairs of enhancer/target-gene in the following categories: Selected enhancer and selected gene (YY), Selected enhancer and non-selected gene (YN), Non-selected enhancer and selected gene (NY), Non-selected enhancer and non-selected gene (NN). A Chi square test is applied to study the dependency of both variables (Chi sqr value = 9.44; p-value = 0.0021).

Chr	Start	End	Length	Test	Population	Closest gene
X	40238664	40241510	2846	iHH12	CEU,GBR,IBS	ATP6AP2
X	45179990	45196717	16727	iHH12	FIN,TSI	KDM6A(*)
X	53740262	53744843	4581	nSL	GWD	HUWE1(**)
X	73135561	73145161	9600	iHS	ESN,GWD,MSL,LWK,YRI	JPX
X	109017803	109018393	590	iHS,nSL	YRI	ACSL4(**)
X	123351438	123353650	2212	iHH12	GWD,MSL,YRI	SH2D1A(**)

Supplementary Table 9. Top enhancer regions under putative positive selection (99.9^{th} percentile). The genes marked as "**" are found under selection in sequence in the 99.9^{th} percentile and in the same continental group, the genes marked as "*" are found under selection as well but in a different continental group.
