# Supplementary material for: Chromosome X-wide Analysis of Positive Selection in Human Populations: Common and Private Signals of Selection and its Impact on Inactivated Genes and Enhancers
Source: Front Genet. 2021 Sep 27;12:714491. doi: 10.3389/fgene.2021.714491 (PMC8502928; doi:10.3389/fgene.2021.714491)
Supplement: Supplementary file 2 [file DataSheet1.PDF]

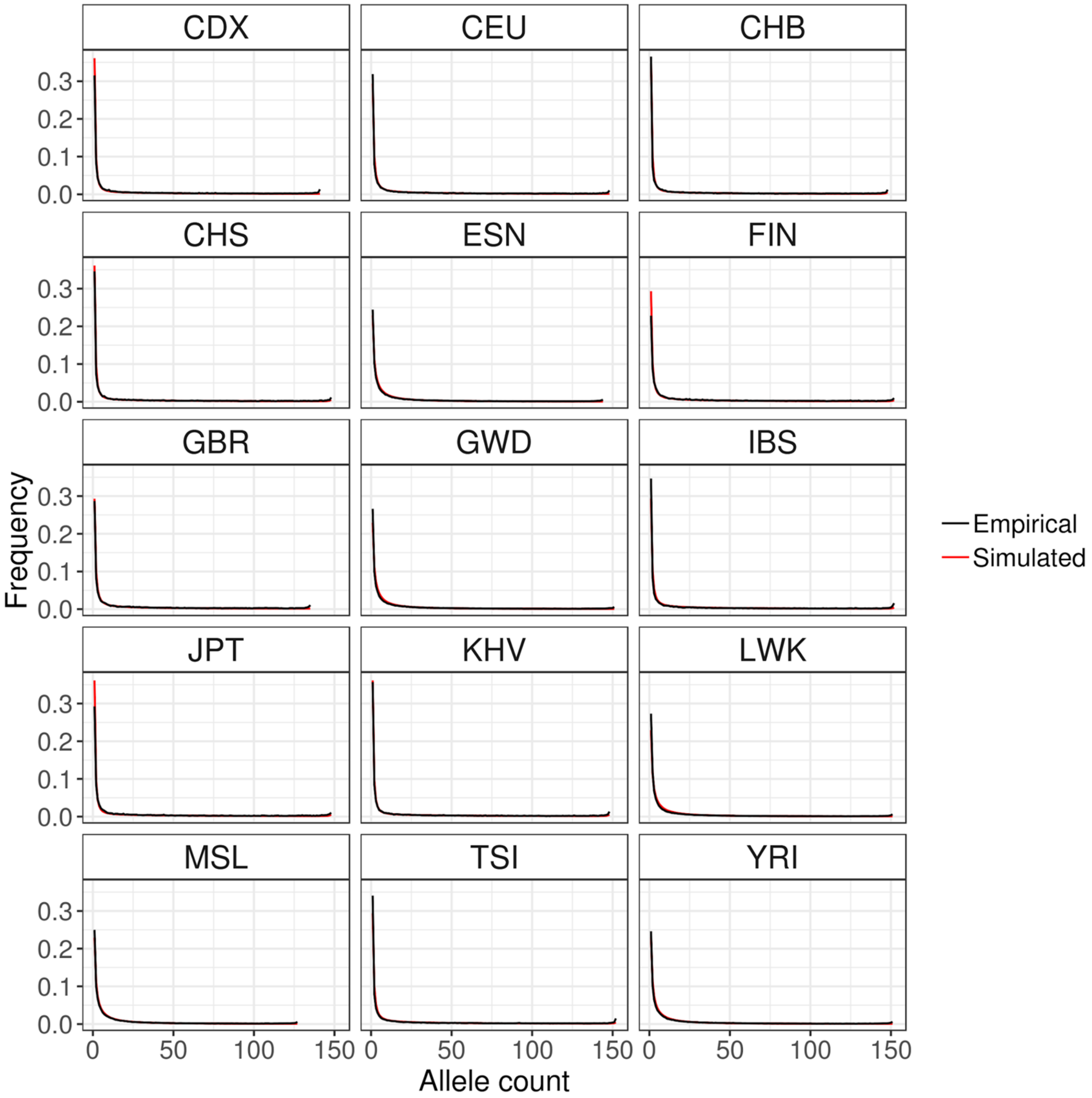

Supplementary Figure 1. Comparison of site frequency spectrums between empirical and simulated data across all populations. Fixed sites have been pruned.

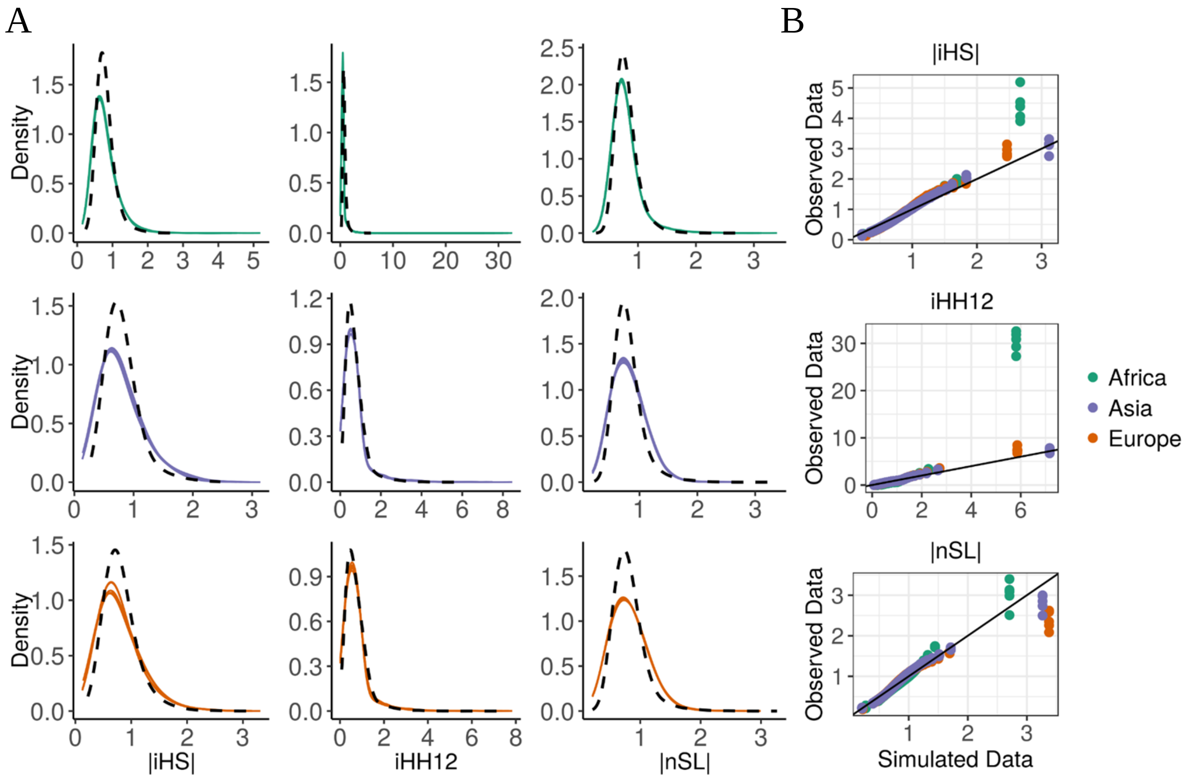

Supplementary Figure 2. A) iHS, iHH12 and nSL distributions (dashed lines as simulated scores) and B) QQ plots of the window-based score distributions in the three geographical groups (Sub-Saharan Africa, Europe, Asia). The QQ plots indicate an overall agreement between the observed and simulated scores. An enrichment of high values in some groups (iHH12 ~ 30) are due to the presence of extreme outliers in the empirical distribution (≥99%), this can be seen in the density plots by the long tail towards positive values.

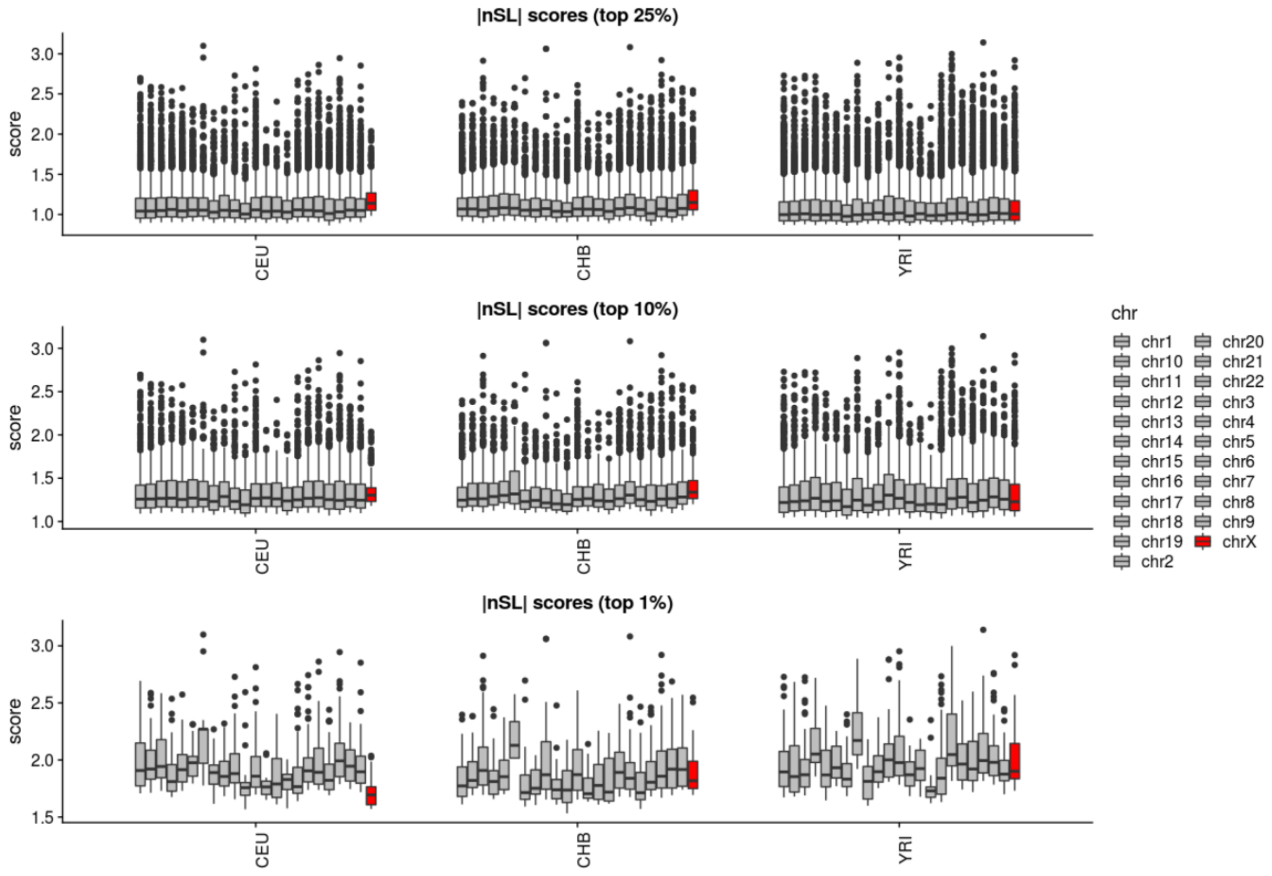

Supplementary Figure 3. Comparison between nSL extreme tail distributions of autosomes and X chromosome.

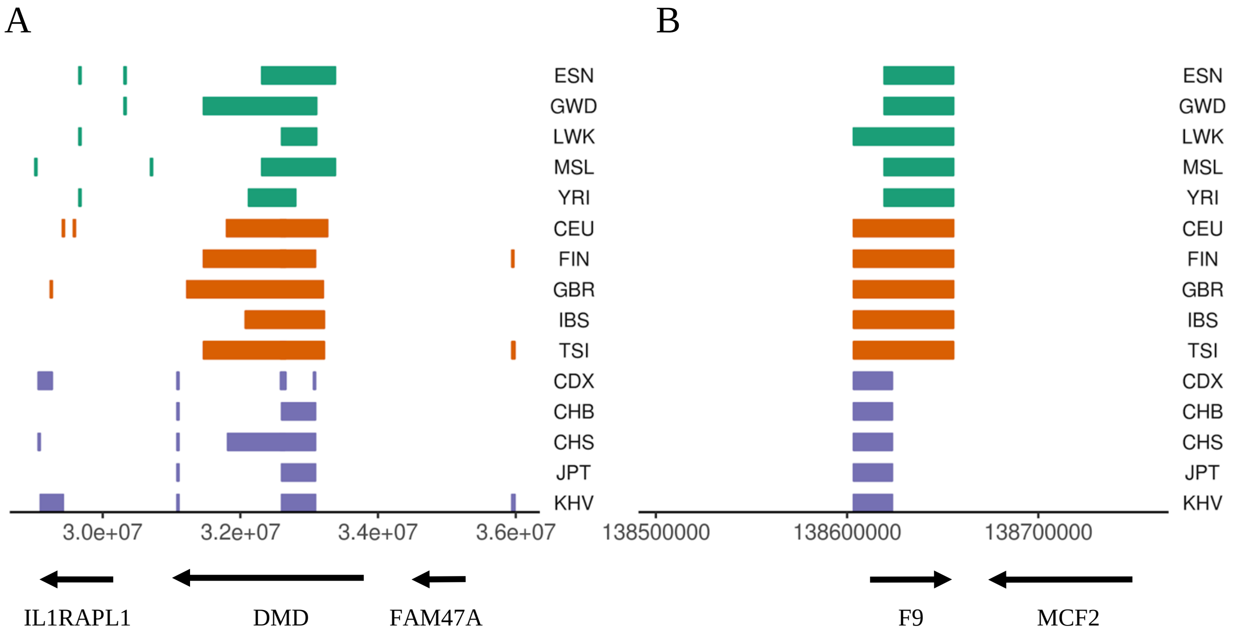

Supplementary Figure 4. Sweeping regions under putative positive selection in the three continental groups in the dystrophin gene (*DMD*; A) and coagulation factor 9 (*F9*; B). The reported sweeps are a result of merging the overlapping windows under positive selection in the 99th in all the statistics used (iHS, iHH12 and nSL).

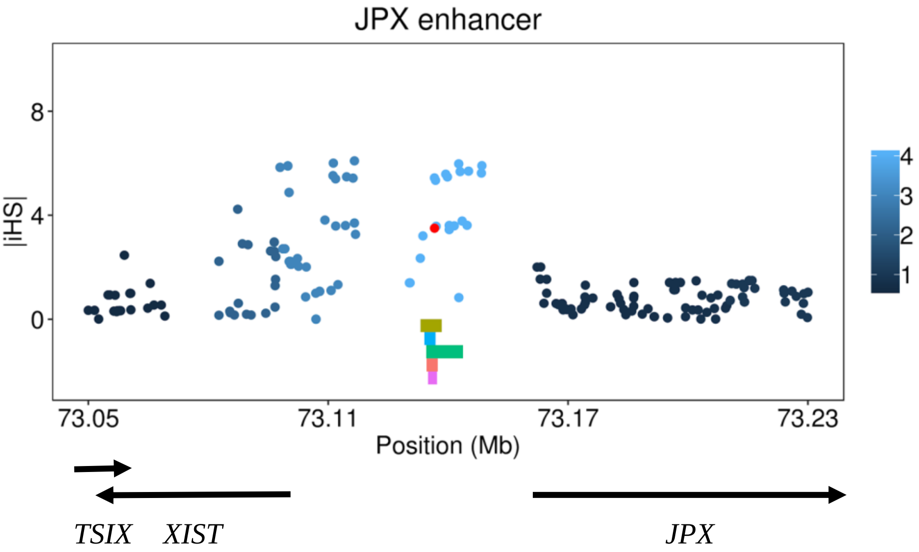

Supplementary Figure 5. Manhattan plot showing the putative positive selection signal in African populations reported by iHS in the enhancer located at ~23kb from the *JPX* gene. Marked in red is the SNP rs112977454 reported as eQTL by GTEx. Colour bars at the bottom represent the active enhancers in five different cell lines. Legend shows the window-based score where the SNPs belong to.
